# The effects of oxygen concentration on cell death, anti-oxidant transcription, acute inflammation, and cell proliferation in precision-cut lung slices

**DOI:** 10.1038/s41598-019-52813-2

**Published:** 2019-11-07

**Authors:** Mitchel J. R. Ruigrok, Jasmine Tomar, Henderik W. Frijlink, Barbro N. Melgert, Wouter L. J. Hinrichs, Peter Olinga

**Affiliations:** 1 University of Groningen, Groningen Research Institute of Pharmacy, Department of Pharmaceutical Technology and Biopharmacy, Antonius Deusinglaan 1, 9713 AV Groningen, The Netherlands; 2 University of Groningen, Groningen Research Institute of Pharmacy, Department of Pharmacokinetics, Toxicology, and Targeting, Antonius Deusinglaan 1, 9713 AV Groningen, The Netherlands; 3 University of Groningen, Groningen Research Institute for Asthma and COPD, Hanzeplein 1, 9713 GZ Groningen, The Netherlands

**Keywords:** Preclinical research, Translational research

## Abstract

Although animal models are often used in drug research, alternative experimental models are becoming more popular as they reduce animal use and suffering. Of particular interest are precision-cut lung slices, which refer to explants – with a reproducible thickness and diameter – that can be cultured *ex vivo*. Because lung slices (partially) reflect functional and structural features of whole tissue, they are often applied in the field of immunology, pharmacology, toxicology, and virology. Nevertheless, previous research failed to adequately address concerns with respect to the viability of lung slices. For instance, the effect of oxygen concentration on lung slice viability has never been thoroughly investigated. Therefore, the main goal of this study was to investigate the effect of oxygen concentration (20 *vs*. 80% O_2_) on the degree of cell death, anti-oxidant transcription, acute inflammation, and cell proliferation in lung slices. According to the results, slices incubated at 20% O_2_ displayed less cell death, anti-oxidant transcription, and acute inflammation, as well as more cell proliferation, demonstrating that these slices were considerably more viable than slices cultured at 80% O_2_. These findings expand our knowledge on lung slices and their use as an alternative experimental model in drug research.

## Introduction

Though animal research is of vital importance in drug research, it is crucial that researchers use alternative experimental models to reduce animal use and suffering. To that end, researchers could use precision-cut lung slices, which are explants – with a reproducible thickness and diameter – that can be cultured *ex vivo*^1^. Advantages of this model include its ability to (partially) reflect the functional and structural heterogeneity of whole tissue and to decrease animal use as more experimental conditions can be tested per animal. Usually, lung slices are made of tissue obtained from guinea pigs, mice, non-human primates, pigs, rats, rabbits, or sheep^2–8^. More importantly, lung slices can also be prepared from human tissue, thereby further expanding the translational value and significance of this model^9^. As a result, lung slices have been used as an experimental model in the field of immunology, pharmacology, toxicology, and virology^10–13^.

In the past, some researchers have reportedly incubated lung slices for as little as just a few hours, although others have cultured lung slices for up to a few weeks^14,15^. Unfortunately, previous research predominantly focused on the application of lung slices as an alternative experimental model but generally failed to accurately address concerns regarding their viability. For example, the effect of oxygen concentration on the viability of lung slices has not been thoroughly investigated. Previously, we cultured lung slices at 80% O_2_, whereas others incubated lung slices at ~20% O_2_^3,16,17^. In addition, slices prepared from different organs (e.g., intestine, liver, and kidney) are typically incubated at 80% O_2_ as well^1,18^. Because a decreasing viability can hamper the use and application of slices as an alternative experimental model, it is important to further investigate whether the oxygen concentration can affect the viability of lung slices.

Therefore, in this study we examined the effect of oxygen concentration (20 *vs*. 80% O_2_) on the degree of cell death, anti-oxidant transcription, acute inflammation, and cell proliferation in lung slices. First, we studied cell death, with a focus on general viability markers (i.e., ATP, protein, and RNA content) as well as tissue damage and DNA fragmentation^19^. Furthermore, we checked whether caspase-dependent apoptosis was triggered by examining accumulation of cleaved CASP3 (cl-CASP3), which is an executioner caspase that can be activated via extrinsic (death signal) or intrinsic (mitochondrial) pathways^20^. Because oxidative stress can lead to cell death, transcription of NRF2-downstream (anti-oxidant) target genes was investigated to estimate whether oxidative stress might have developed in lung slices^21^. In addition, to characterize acute inflammation, we analyzed mRNA expression of pro-inflammatory cytokines as well as the release of respective proteins into culture medium^22^. Finally, cell proliferation in slices was assessed by measuring mRNA expression of proliferation-related genes and by staining lung slices for Ki67 – a marker of proliferation^23^.

## Materials and Methods

### Animal tissue

Lung tissue was obtained from male C57BL/6 J mice (10-14 weeks old; 24-30 gram), which were housed under controlled conditions with a 12 h light/dark cycle and *ad libitum* access to water and food (Central Animal Facility, University Medical Center Groningen, Groningen, The Netherlands). Mice were anesthetized with isoflurane/O_2_ (Nicolas Piramal, London, UK) and sacrificed by exsanguination via the inferior vena cava followed by perforation of the diaphragm. Directly afterwards, lungs were inflated *in situ* with 1 mL of liquefied and pre-warmed (37 °C) support medium containing 1.5% low-gelling-temperature agarose (Sigma-Aldrich, Zwijndrecht, The Netherlands) and 0.9% NaCl (Merck, Darmstadt, Germany). After inflation and excision, lungs were immediately transferred to ice-cold University of Wisconsin preservation solution (DuPont Critical Care, Waukegab, USA). The animal experiments were approved by the Central Authority for Scientific Procedures on Animals (permit number: 20171290) and conducted conform criteria set out in national and international legislation.

### Precision-cut lung slices

Slices (wet weight of 4-5 mg; thickness of 250-350 μm; diameter of 5 mm) were prepared with a Krumdieck tissue slicer (Alabama Research and Development, Munford, USA), which was filled with ice-cold Krebs-Henseleit buffer supplemented with 25 mM D-glucose (Merck), 25 mM NaHCO_3_ (Merck), and 10 mM HEPES (MP Biomedicals, Aurora, USA); saturated with carbogen gas (95% O_2_ and 5% CO_2_); and adjusted to a pH of 7.4^3^. After slicing, slices were sampled directly (0 h) or pre-incubated in 12-well plates (1 slice/well) containing 1 mL/well of pre-warmed (37 °C) PneumaCult-ALI culture medium (Stemcell Technologies, Grenoble, France), which was supplemented with 100 U/mL penicillin-streptomycin (Life Technologies, Bleiswijk, The Netherlands) and 50 μg/mL gentamicin (Life Technologies), at 5% CO_2_ and either 20 or 80% O_2_. Culture plates were gently shaken at 90 cycles/min. After a pre-incubation of 2 h, slices were transferred to culture plates with fresh and prewarmed culture medium and they were incubated for 48 or 96 h, after which samples were taken. Culture medium of slices that were incubated for 96 h was refreshed after 48 h.

### ATP/protein content

Intracellular adenosine triphosphate (ATP) was extracted from slices (3 per condition), which were individually stored in 1 mL of ice-cold sonication solution (70% ethanol and 2 mM EDTA) at −80 °C, as previously described^3^. Briefly, slices were homogenized using a Minibead-beater (2 cycles of 45 s) and subsequently centrifuged (16,000 × *g* at 4 °C for 5 min). The obtained supernatant was analyzed with an ATP Bioluminescence Kit (Roche Diagnostics, Mannheim, Germany). Calculated ATP values (pmol) were then normalized to the total amount of protein (μg), which was determined for individual slices using an RC DC Protein Assay (Bio-Rad, Munich, Germany).

### Cytokine release

Culture medium samples (from 3 wells) were analyzed with a Mouse IL-1β DuoSet enzyme-linked immunosorbent assay (ELISA), Mouse IL-6 DuoSet ELISA, and Mouse TNF-α DuoSet ELISA (Bio-Techne, Abingdon, UK), according to the manufacturer’s instructions. Optical densities were measured with a BioTek Synergy HT (BioTek Instruments, Vermont, USA). To correct for optical imperfections in the plate, wavelength correction was applied by subtracting readings at 540 nm from readings at 450 nm.

### mRNA expression

Total RNA was extracted from slices (6 per condition) with a Maxwell 16 LEV SimplyRNA Tissue Kit (Promega, Leiden, The Netherlands), after which the RNA yield and purity was quantified using a NanoDrop ND-100 spectrophotometer (NanoDrop Technologies, Wilmington, USA). Next, the extracted RNA was reverse transcribed with a Reverse Transcription System Kit (Promega) and thermal cycler (22 °C for 10 min, 42 °C for 15 min, and 95 °C for 5 min). Thereafter, the real-time quantitative polymerase chain reaction (qPCR) analysis was conducted with specific primers (Table 1), FastStart Universal SYBR Green Master Mix (Roche, Almere, The Netherlands), and a ViiA7 real-time qPCR (Applied Biosystems, Bleiswijk, The Netherlands), using 1 cycle of 10 min at 95 °C and 40 consecutive cycles of 15 s at 95 °C, 30 s at 60 °C, and 30 s at 72 °C. mRNA expression was calculated as fold induction, using *Ywhaz* as a reference gene.

**Table 1:** Primers.

Gene	Forward sequence (5′ → 3′)	Reverse sequence (5′ → 3′)
*Casp3*	ATGGAGAACAACAAAACCTCAGT	TTGCTCCCATGTATGGTCTTTAC
*Ccna2*	AAGAGAATGTCAACCCCGAAAAA	ACCCGTCGAGTCTTGAGCTT
*Ccnb1*	CTTGCAGTGAGTGACGTAGAC	CCAGTTGTCGGAGATAAGCATAG
*Ccnd1*	GCGTACCCTGACACCAATCTC	ACTTGAAGTAAGATACGGAGGGC
*Ccne1*	CTCCGACCTTTCAGTCCGC	CACAGTCTTGTCAATCTTGGCA
*Gclc*	GGGGTGACGAGGTGGAGTA	GTTGGGGTTTGTCCTCTCCC
*Gclm*	AGGAGCTTCGGGACTGTATCC	GGGACATGGTGCATTCCAAAA
*Hmox1*	AAGCCGAGAATGCTGAGTTCA	GCCGTGTAGATATGGTACAAGGA
*Il1b*	TGAGCACCTTCTTTTCCTTCA	TTGTCTAATGGGAACGTCACAC
*Il6*	TGATGCTGGTGACAACCACGGC	TAAGCCTCCGACTTGTGAAGTGGTA
*Mki67*	ATCATTGACCGCTCCTTTAGGT	GCTCGCCTTGATGGTTCCT
*Nqo1*	AGGATGGGAGGTACTCGAATC	AGGCGTCCTTCCTTATATGCTA
*Srxn1*	CCCAGGGTGGCGACTACTA	GTGGACCTCACGAGCTTGG
*Tnfa*	CTGTAGCCCACGTCGTAGC	TTGAGATCCATGCCGTTG
*Txnrd1*	CCCACTTGCCCCAACTGTT	GGGAGTGTCTTGGAGGGAC
*Ywhaz*	TTACTTGGCCGAGGTTGCT	TGCTGTGACTGGTCCACAAT

### Protein expression

Protein was isolated from slices (6 per condition), using ice-cold RIPA lysis buffer (Fischer Scientific, Landsmeer, The Netherlands) and a Minibead-beater for homogenization (five cycles of 45 s Minibead-beating and 10 min of cooling on ice), as described previously^16^. After centrifugation of the lysate (16,000 × *g* at 4 °C for 30 min), the supernatant was collected and analyzed to determine the protein concentration. Samples were subsequently boiled (100 °C for 15 min) to denature protein. Thereafter, protein (20 μg) was separated through sodium dodecyl sulfate-polyacrylamide gel electrophoresis (SDS-PAGE), using 10% gels, and blotted onto polyvinylidene fluoride membranes using a Trans-Blot Turbo Transfer System (Bio-Rad). Afterwards, membranes were blocked in 5% non-fat milk/TBST (Bio-Rad) for 1 h, after which they were incubated overnight with primary antibody (Table 2) at 4 °C, followed by incubation with the respective secondary antibody for 1 h. Finally, protein was visualized with Clarity Western ECL blotting substrate (Bio-Rad) using the ChemiDoc Touch Imaging System (Bio-Rad). Protein expression was normalized against vinculin (VCL), which was used as a loading control.

**Table 2:** Antibodies.

Protein	Primary antibody	Secondary antibody
cl-CASP3	Rabbit anti-CASP3 (cleaved form) (ab13847, 1:500, Abcam, Cambridge, USA)	Goat anti-rabbit HRP (P0448, 1:2000, Dako, Santa Clara, USA)
VCL	Mouse anti-VCL (sc-73614, 1:500, Santa Cruz, California, USA)	Rabbit anti-mouse HRP (P0260, 1:5000, Dako)

### Stainings

Slices (3 per condition) were fixed in 4% formalin at 4 °C for 24 h, after which they were dehydrated in baths with increasing strengths of ethanol. Next, slices were cleared in xylene baths and embedded horizontally in paraffin. Before staining, sections (4 μm) were deparaffinized and rehydrated in baths with decreasing strengths of ethanol. Tissue damage was visualized with a routine hematoxylin and eosin (H&E) staining, whereas DNA fragmentation was revealed with a terminal deoxynucleotidyl transferase dUTP nick end labeling (TUNEL) Assay HRP-DAB Kit (Abcam). Furthermore, proliferation was assessed with an immunohistochemical staining for Ki67. Briefly, sections were subjected to heat-mediated (80 °C) antigen retrieval in 10 mM Tris/1 mM EDTA (pH 9.0) for 15 min, blocked in 2% BSA/2% human serum in PBS, incubated with rabbit anti-Ki67 (ab15580, 1:750, Abcam) for 1 h, washed with 0.05% Tween 20 in PBS (3 times for 5 min), incubated with goat anti-rabbit HRP (P0160, 1:50, Dako) for 30 min, washed with 0.05% Tween 20 in PBS (3 times for 5 min), incubated with ImmPACT NovaRED Peroxidase Substrate (VectorLabs, Burlingame, USA) for 5 min, counterstained with hematoxylin for 1-2 seconds, and mounted with DEPEX (Sigma-Aldrich). Stained sections were scanned with a C9600 NanoZoomer (Hamamatsu Photonics, Hamamatsu, Japan). Semi-quantitative tissue damage scores were assigned to the airways and parenchyma in a blinded manner by two independent observers, using a custom scoring system (supplementary information, section 1). DNA fragmentation and proliferation were quantified with Aperio ImageScope (V12.3.3), employing the built-in Aperio Positive Pixel Count algorithm (V9). For each sample (biological replicate), three entire slices (technical replicates) were analyzed using the default algorithm settings to count negative, weak positive, medium positive, and strong positive pixels in scanned whole-slide images. To avoid inclusion of pixels reflecting non-specific staining, the staining intensity was calculated as the ratio of strong positive pixels *vs*. total pixels (supplementary information, section 2).

### Statistics

GraphPad Prism (version 6.0) was used to analyze data using two-way analyses of variance followed by Bonferroni’s post-hoc test to compare means between incubation conditions at specific timepoints. Differences between groups were considered to be statistically significant when *p* < 0.05.

## Results

### Characterization of general viability markers

**Figure 1:**
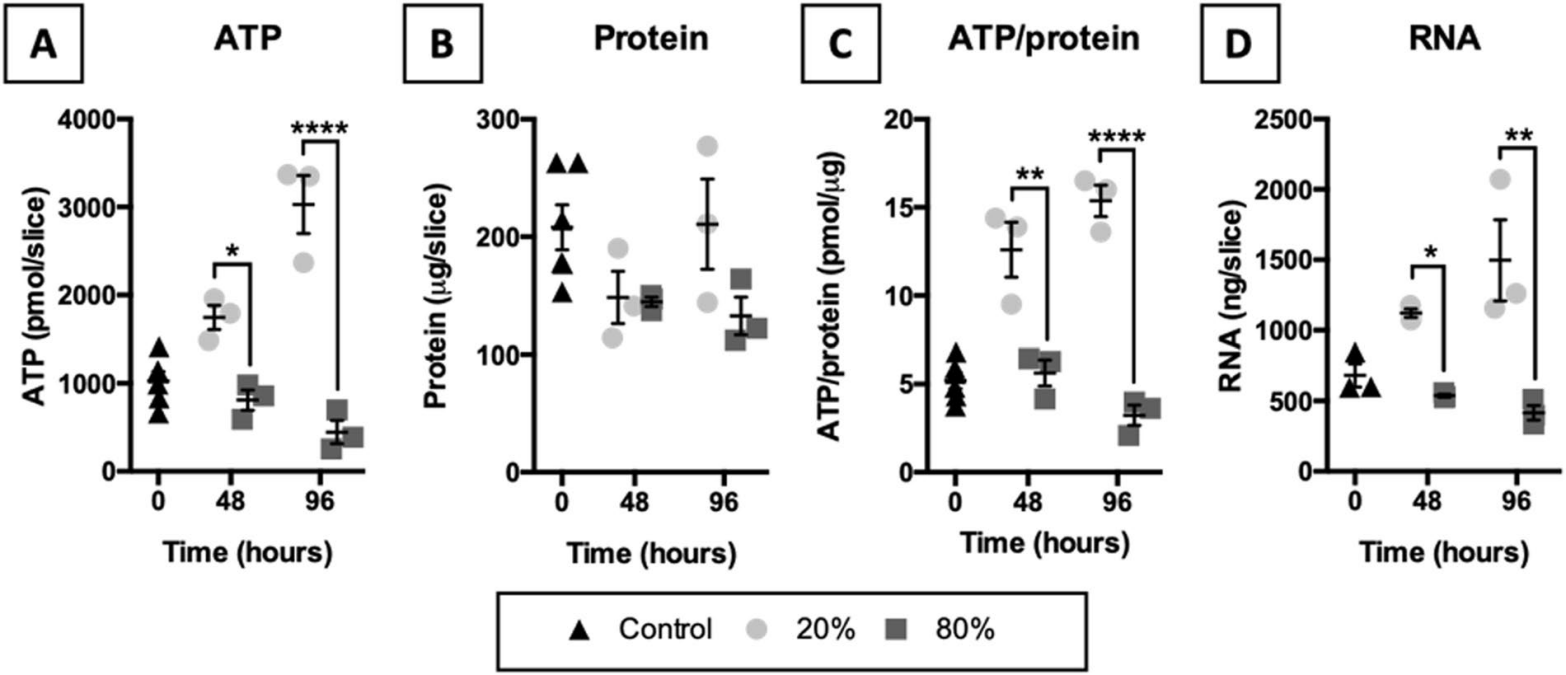
Characterization of general viability markers. Slices were collected after slicing (0 h) and after 48 or 96 h of incubation at either 20 or 80% O_2_ (*n* = 3-6). ATP (**A**), protein (**B**), ATP/protein (**C**), and RNA (**D**) content in slices was measured to identify potential changes in viability. Values represent individual experiments performed in triplicate and are accompanied with the arithmetic mean (horizontal line) ± standard error of the mean (error bars). (*p < 0.05, **p < 0.01, and ****p < 0.0001).

To identify potential changes in viability, we quantified ATP, protein, and RNA content in slices (Fig. 1). According to the results, exposure to 20% O_2_ elevated the ATP, ATP/protein, and RNA content in slices over time. In contrast, these parameters gradually declined in slices that were incubated at 80% O_2_. The protein content, however, remained similar between the tested oxygen concentrations.

### Evaluation of tissue damage

**Figure 2:**
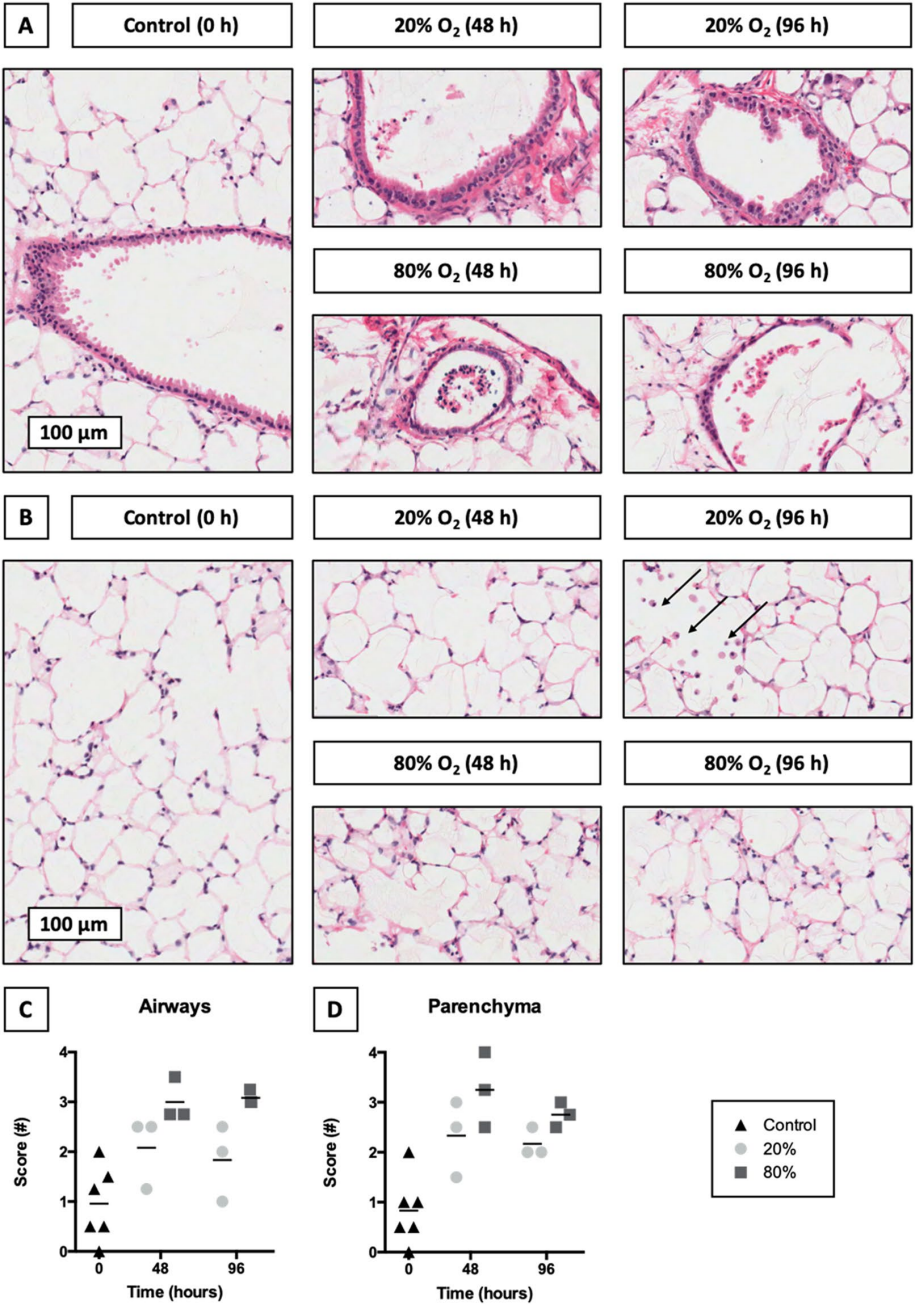
Evaluation of tissue damage. Samples were taken after slicing (0 h) and after 48 or 96 h of incubation at either 20 or 80% O_2_ (*n* = 3-6). HE stainings were carried out to study tissue damage in the airways (**A**) and parenchyma (**B**). Arrows point towards macrophages. In addition, tissue damage scores were assigned to the airways (**C**) and parenchyma (**D**). Scores ranged from 0 (no tissue damage) to 4 (severe tissue damage). Values represent an average of the scores assigned by two independent observers and are accompanied with the arithmetic mean (horizontal line).

To reveal potential tissue damage in the airways and parenchyma, we conducted an HE staining (Fig. 2). As shown, slices incubated at 20% O_2_ displayed fewer signs of tissue damage than slices cultured at 80% O_2_. With respect to the airways, the epithelium generally maintained its pseudostratified structure at 20% O_2_. After exposure to 80% O_2_, however, epithelial cells detached from the basement membrane, leading to the flattening of remaining cells. Substantial differences in tissue damage were also observed in the parenchyma. More specifically, hyperoxic incubation conditions (80% O_2_) led to more extensive karyolysis (nuclei dissolution), pyknosis (nuclei shrinkage), and karyorrhexis (nuclei fragmentation) in the parenchyma. Furthermore, alveolar macrophages were abundant in slices cultured for 96 h at 20% O_2_ but not in slices cultured at 80% O_2_.

### Localization of DNA fragmentation

**Figure 3:**
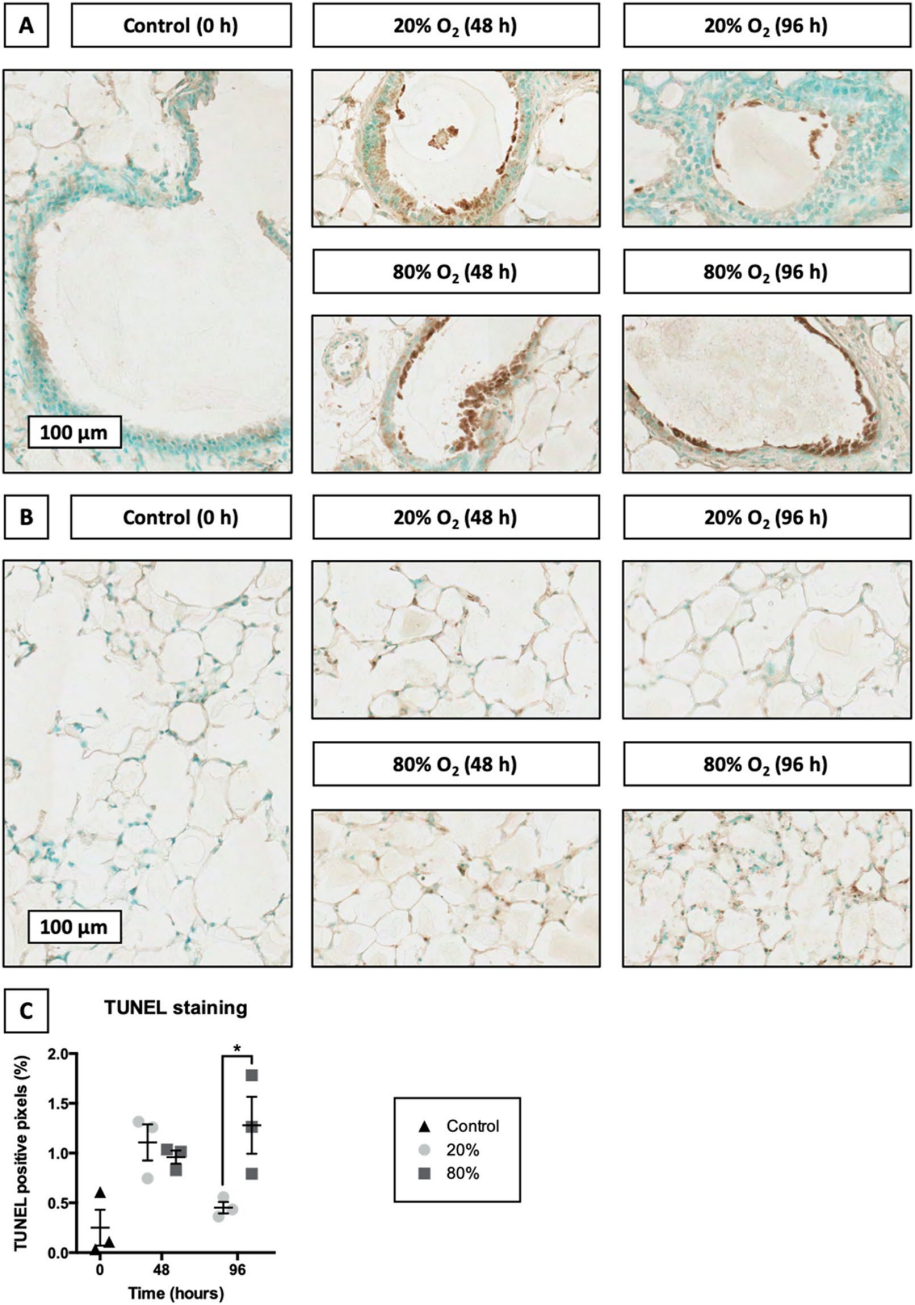
Localization of DNA fragmentation. Slices were sampled after slicing (0 h) and after 48 or 96 h of incubation at either 20 or 80% O_2_ (*n* = 3). TUNEL stainings were performed to assess DNA fragmentation in the airways (**A**) and parenchyma (**B**). Subsequent algorithmic analysis revealed TUNEL positive pixels (**C**). Values represent individual experiments performed in triplicate and are accompanied with the arithmetic mean (horizontal line) ± standard error of the mean (error bars). (*p < 0.05).

To investigate cell death in slices, we performed TUNEL stainings, which can be used to reveal DNA fragmentation – a hallmark sign of apoptosis and necrosis (Fig. 3). As illustrated, DNA fragmentation became considerably more apparent in slices after 48 h of incubation, regardless of the oxygen concentration. However, after 96 h of incubation at 20% O_2_, the degree of DNA fragmentation in slices declined to original levels, whereas it continued to increase in slices cultured at 80% O_2_. Furthermore, instances of DNA fragmentation were spread uniformly throughout the entire slice and marked both the airways and parenchyma.

### Activation of caspase-dependent apoptosis

**Figure 4:**
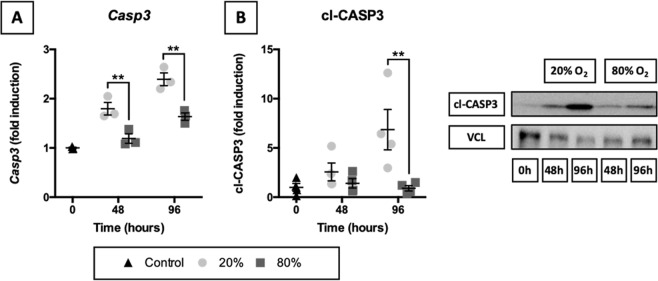
Activation of caspase-dependent apoptosis. Samples were obtained after slicing (0 h) and after 48 or 96 h of incubation at either 20 or 80% O_2_ (*n* = 3–4). Baseline mRNA expression of *Casp3* (**A**) was examined via qPCR. Cleavage of CASP3 (**B**) was investigated by western blotting, using vinculin (VCL) as a loading control. Values represent individual experiments performed in triplicate and are accompanied with the arithmetic mean (horizontal line) ± standard error of the mean (error bars). (**p < 0.01).

To check whether caspase-dependent apoptosis was triggered in slices, we examined *Casp3* mRNA expression and accumulation of its functional protein cl-CASP3 (Fig. 4). Even though caspase-dependent apoptosis is regulated on a post-translational level by cleavage of CASP3, we measured *Casp3* mRNA levels to determine whether its expression was strongly up- or downregulated in slices during incubation. As illustrated, both *Casp3* mRNA and cl-CASP3 became gradually more abundant in slices incubated at 20% O_2_. In contrast, slices cultured at 80% O_2_ displayed negligible changes in these parameters.

### Transcription of NRF2-downstream target genes

**Figure 5:**
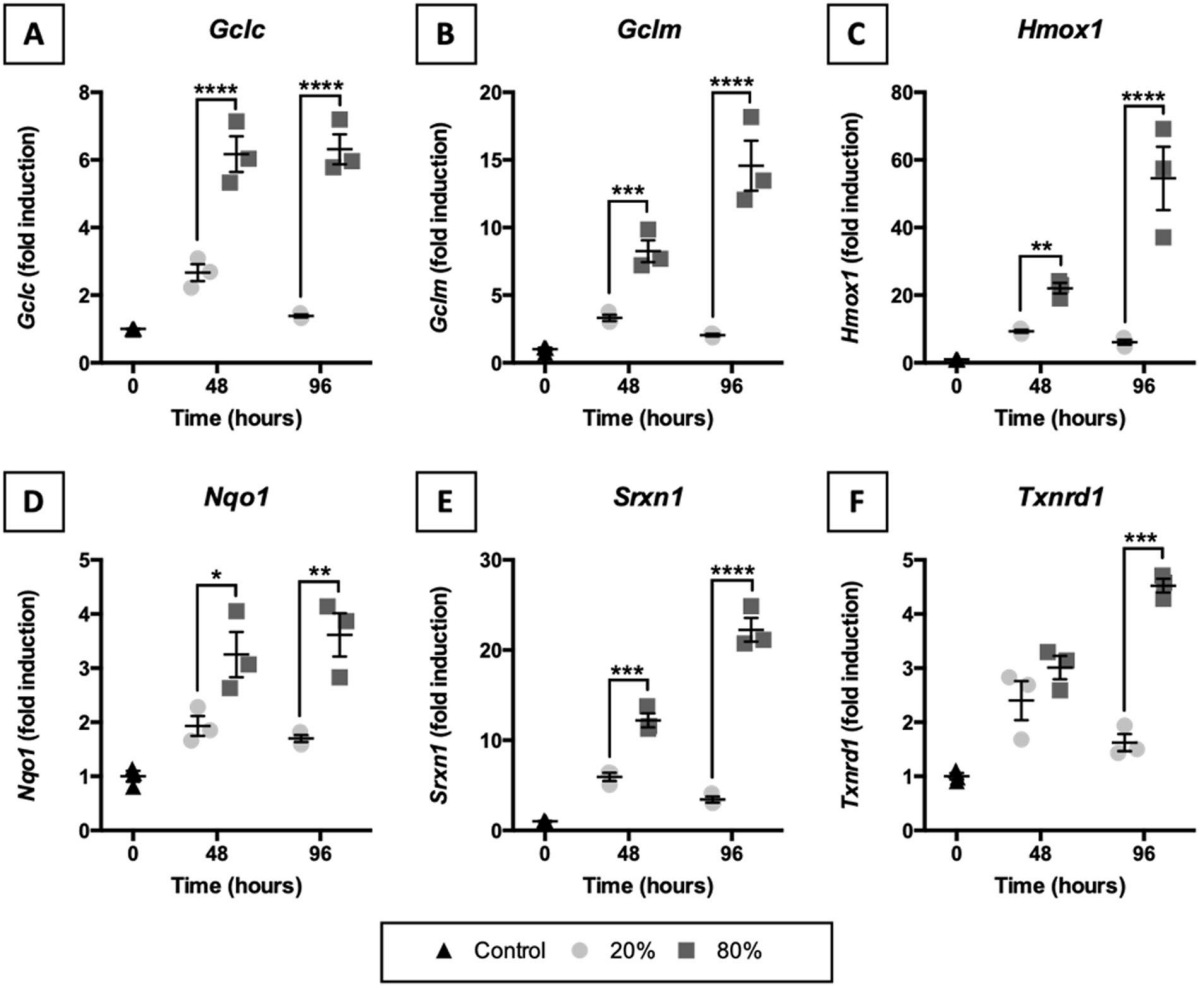
Transcription of NRF2-downstream target genes. Slices were sampled after slicing (0 h) and after 48 or 96 h of incubation at either 20 or 80% O_2_ (*n* = 3). qPCR was used to analyze *Gclc* (**A**), *Gclm* (**B**), *Hmox1* (**C**), *Nqo1* (**D**), *Srxn1* (**E**), and *Txnrd1* (**F**) mRNA expression because they are NRF2-downstream target genes. Values represent individual experiments performed in triplicate and are accompanied with the arithmetic mean (horizontal line) ± standard error of the mean (error bars). (*p < 0.05, **p < 0.01, ***p < 0.001, and ****p < 0.0001).

To estimate whether oxidative stress might have developed in slices, we studied mRNA expression of NRF2-downstream target genes (Fig. 5). As demonstrated, NRF2-downstream target genes were clearly transcribed more profusely in slices cultured at 80% O_2_. Transcription of NRF2-downstream target genes increased after 48 h of incubation, albeit to a different extent depending on the oxygen concentration. Interestingly, after 96 h of incubation at 20% O_2_, mRNA expression of NRF2-downstream target genes either leveled off or declined, whereas mRNA expression continued to increase in slices that were cultured at 80% O_2_.

### Expression of pro-inflammatory cytokines

**Figure 6:**
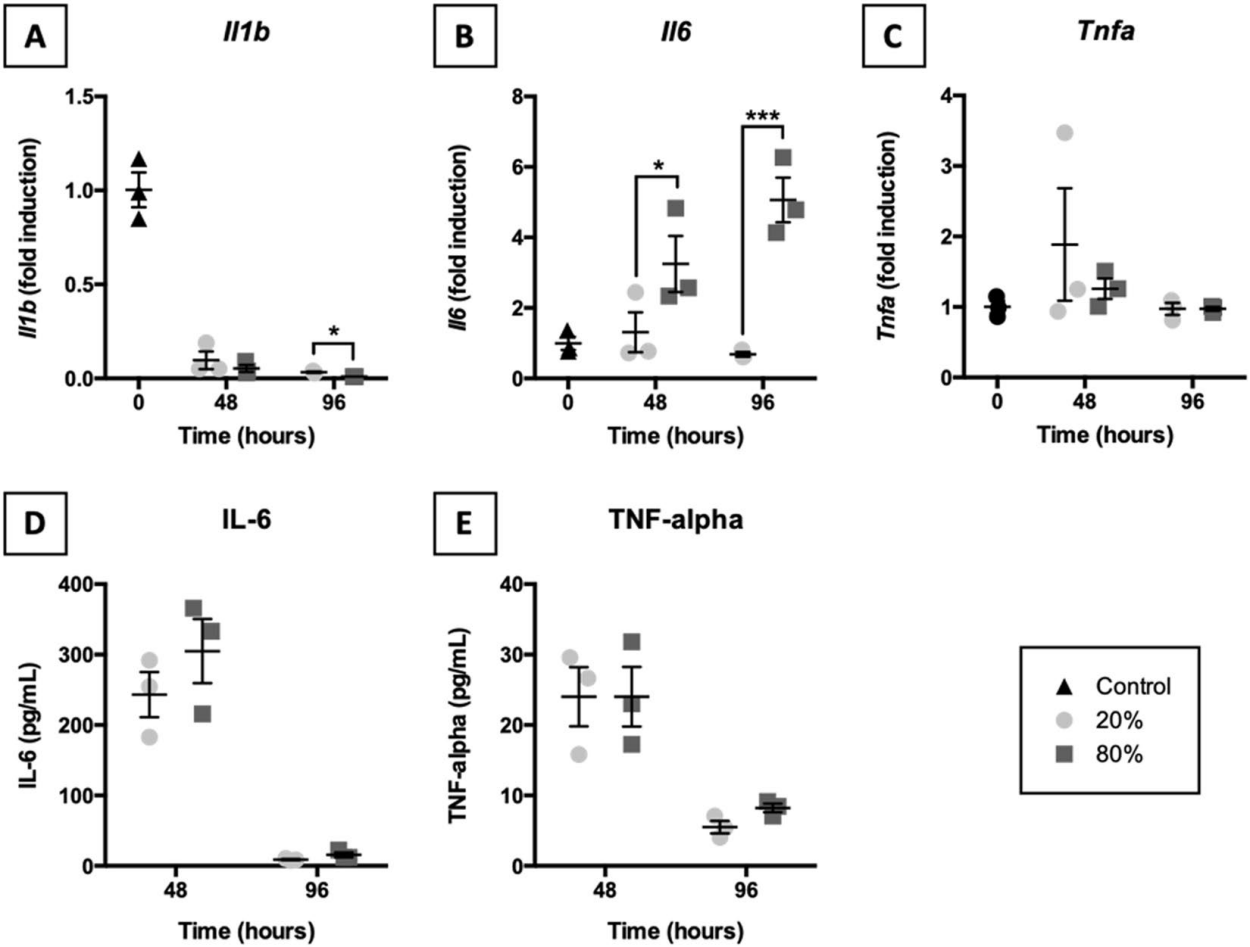
Expression of pro-inflammatory cytokines. Samples were taken after slicing (0 h) and after 48 or 96 h of incubation at either 20 or 80% O_2_ (*n* = 3). qPCR was used to assess *Il1b* (**A**), *Il6* (**B**), and *Tnfa* (**C**) mRNA expression. Release of IL-6 (**D**) and TNF-α (**E**) into culture medium was quantified by ELISA. Values represent individual experiments performed in triplicate and are accompanied with the arithmetic mean (horizontal line) ± standard error of the mean (error bars). (*p < 0.05 and ***p < 0.001).

To establish whether acute inflammation manifested in slices, we measured *Il1b*, *Il6*, and *Tnfa* mRNA expression and release of respective proteins (i.e., IL-1β, IL-6, and TNF-α) into culture medium (Fig. 6). As shown, *Il1b* mRNA expression strongly decreased over time regardless of the oxygen concentration, whereas *Tnfa* mRNA expression remained unchanged. mRNA expression of *Il6*, however, appeared to be affected by oxygen levels because it was elevated in slices exposed to 80% O_2_. In addition, time-dependent differences were observed in the release of IL-6 and TNF-α into culture medium, but no oxygen-dependent effects were discovered (IL-1β was not detectable). Release of IL-6 and TNF-α occurred mainly during the first 48 h of incubation, after which it declined again.

### Induction of proliferation-related genes

**Figure 7:**
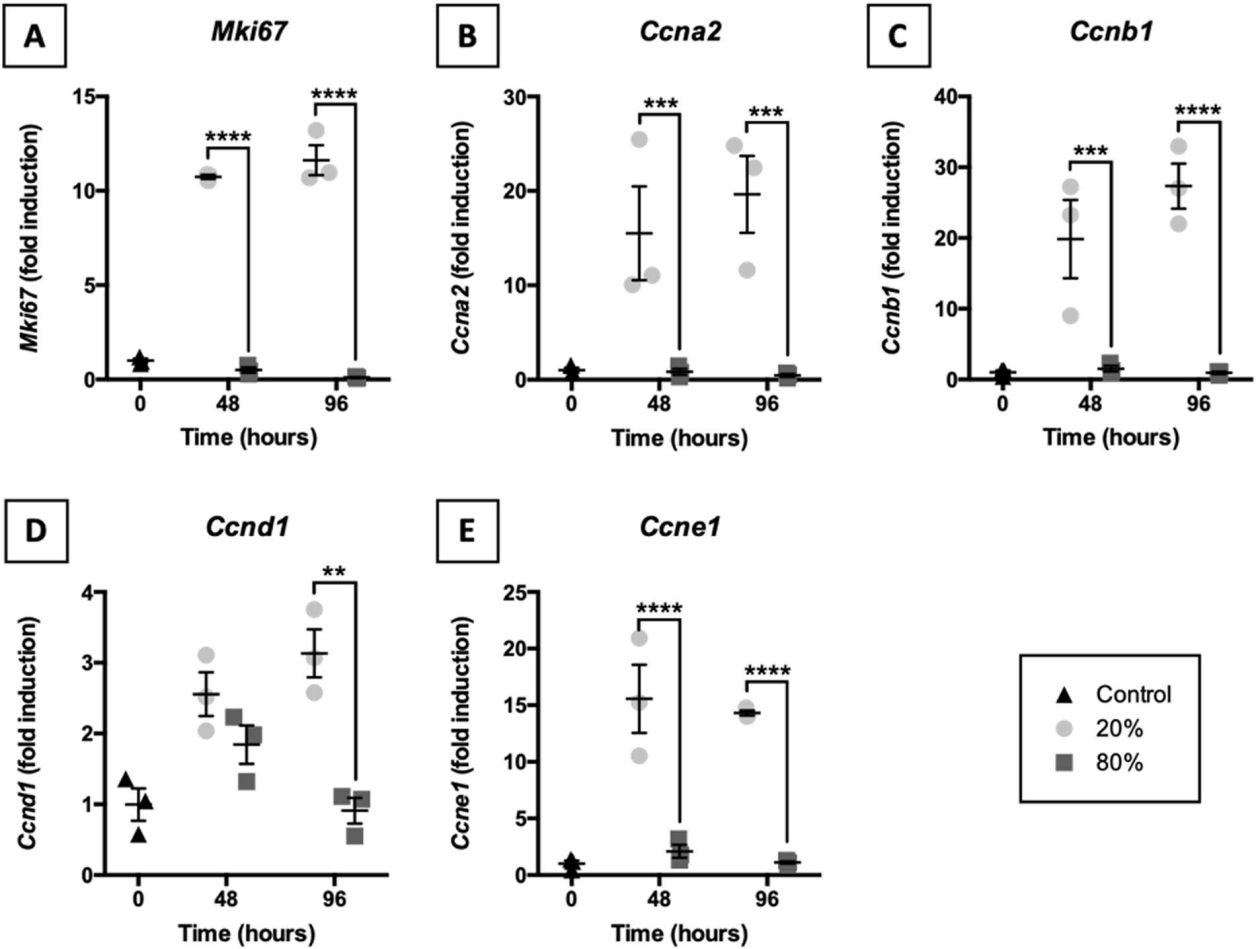
Induction of proliferation-related genes. Slices were collected after slicing (0 h) and after 48 or 96 h of incubation at either 20 or 80% O_2_ (*n* = 3). qPCR was also used to study mRNA expression of proliferation-related genes *Mki67* (**A**), *Ccna2* (**B**), *Ccnb1* (**C**), *Ccnd1* (**D**), and *Ccne1* (**E**). Values represent individual experiments performed in triplicate and are accompanied with the arithmetic mean (horizontal line) ± standard error of the mean (error bars). (**p < 0.01, ***p < 0.001, and ****p < 0.0001).

To explore the induction of proliferation in slices, we assessed mRNA expression of proliferation-related genes (i.e., *Mki67*, *Ccna2*, *Ccnb1*, *Ccnd1*, and *Ccne1*) (Fig. 7). Distinct expression patterns were observed for slices incubated at 20 and 80% O_2_. Exposure of slices to 20% O_2_ resulted in a strongly increased expression of the previously mentioned genes. This observation was not made for slices cultured at 80% O_2_. Instead, at 80% O_2_, mRNA expression of proliferation-related genes remained relatively unchanged or diminished over time.

### Localization of proliferating cells

**Figure 8:**
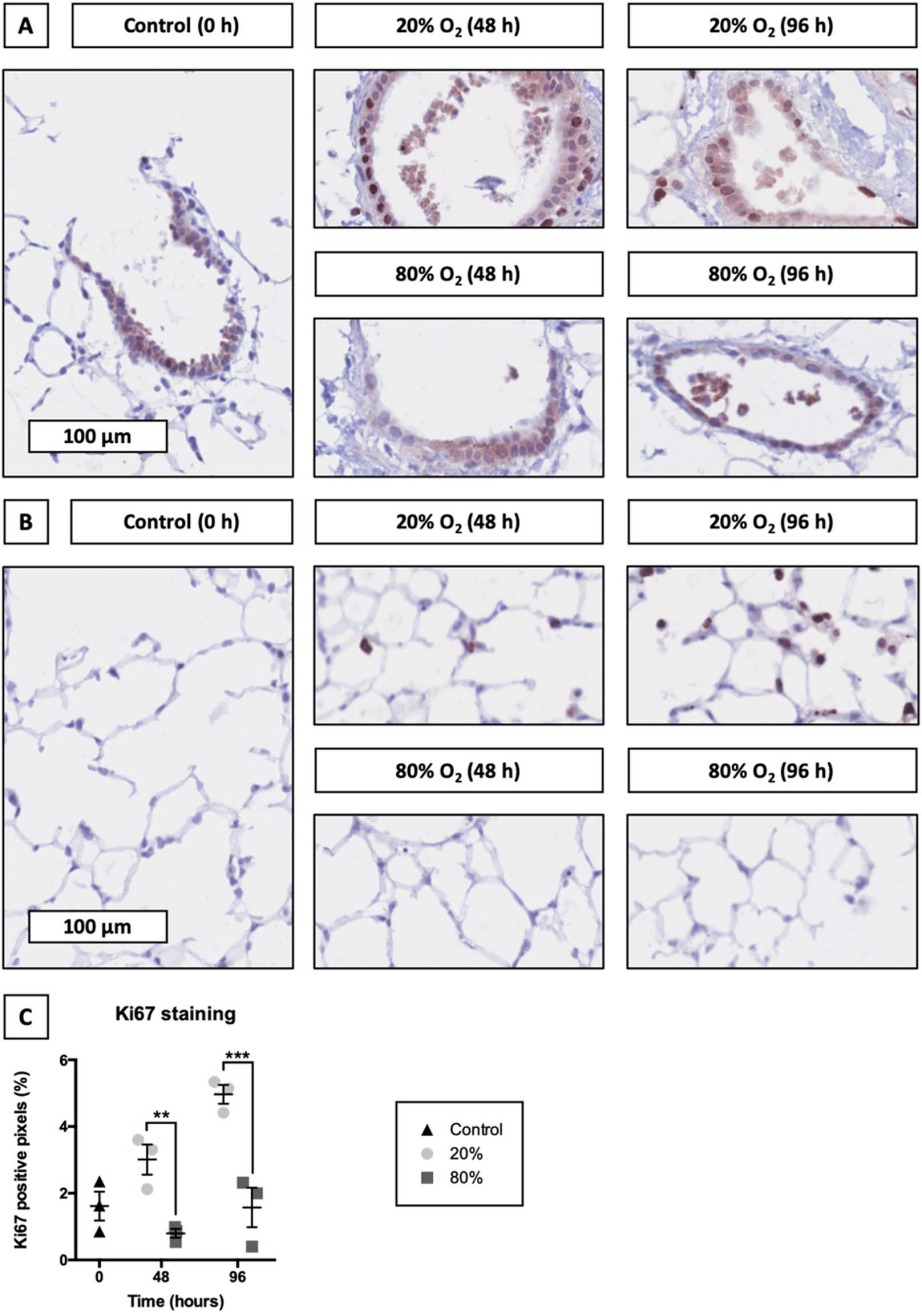
Localization of proliferating cells. Samples were obtained after slicing (0 h) and after 48 or 96 h of incubation at either 20 or 80% O_2_ (*n* = 3). Ki67 stainings were performed to localize proliferating cells in the airways (**A**) and parenchyma (**B**). Subsequent algorithmic analysis revealed Ki67 positive pixels (**C**). Values represent individual experiments performed in triplicate and are accompanied with the arithmetic mean (horizontal line) ± standard error of the mean (error bars). (**p < 0.01 and ***p < 0.001).

To localize proliferating cells in slices, we stained tissue sections for Ki67 – a protein that is expressed in proliferating cells (Fig. 8). As displayed, profound differences were observed in the Ki67 staining intensity between slices incubated at 20 and 80% O_2_. Incubating slices at 20% O_2_ led to a progressive increase in the Ki67 staining intensity, which was 3-fold lower in slices cultured at 80% O_2_. Although Ki67 was detected throughout the entire slice when cultured at 20% O_2_, at 80% O_2_ Ki67 remained mostly confined to the airways and was only sporadically observed in the parenchyma.

## Discussion

The main objective of this study was to investigate the effect of oxygen concentration (20 *vs*. 80% O_2_) on the extent of cell death, anti-oxidant transcription, acute inflammation, and cell proliferation in lung slices. Our study provides considerable insights into the effect of oxygen concentration on the viability of lung slices. More specifically, lung slices cultured at 20% O_2_ displayed less cell death, anti-oxidant transcription, and acute inflammation, as well as more cell proliferation, demonstrating these slices were significantly more viable than slices incubated at 80% O_2_.

First of all, overall changes in viability were identified by measuring ATP, protein, and RNA content in slices. Remarkably, the ATP and RNA content gradually increased in slices that were incubated at 20% O_2_, whereas these parameters progressively declined in slices cultured at 80% O_2_ (protein content remained unaffected). The decline in ATP content suggests mitochondrial function was severely hampered in slices exposed to 80% O_2_. Severe mitochondrial impairment typically leads to a switch from apoptosis, which is an energy-requiring process, to necrosis and an inflammatory response^20^. In addition, ATP depletion can activate AMPK, thereby leading to subsequent activation of P53, which can induce either apoptosis or cell-cycle arrest^24^. Furthermore, the decline in RNA content also indicates more cell death occurred in slices exposed to 80% O_2_ because RNA is rapidly degraded during necrosis and apoptosis^25^. In contrast, the ATP and RNA content in slices incubated at 20% O_2_ could have increased due to cell proliferation, which requires ATP in order to replicate cellular contents^26^.

We subsequently stained sections with H&E to reveal potential tissue damage in lung slices. In general, slices incubated at 20% O_2_ exhibited fewer signs of tissue damage than slices cultured at 80% O_2_. Furthermore, tissue damage was already observed after 48 h of incubation, after which it leveled off. For example, tissue damage in the airways was predominantly characterized by the detachment of perishing epithelial cells from the basement membrane, while remaining epithelial cells flattened to cover the denuded surface. These features are usually observed in response to injury of the airways^27^. Tissue damage in the parenchyma was marked by instances of pyknosis, karyolysis, and karyorrhexis. In addition, apoptotic bodies were particularly prominent in slices cultured at 80% O_2_. These findings are in agreement with our previous study where we also observed signs of tissue damage in slices cultured at 80% O_2_^16^. Alveolar macrophages were only abundant in slices cultured at 20% O_2_ for 96 h. This indicates that the viability of alveolar macrophages can be affected by the oxygen concentration.

Thereafter, TUNEL stainings were used to evaluate DNA fragmentation, which is a clear sign of cell death^19^. Interestingly, after 48 h of incubation, slices cultured at either 20 or 80% O_2_ displayed a comparable increase in the extent of DNA fragmentation. This initial increase suggests cell death was a result of mechanical and/or chemical stress that slices were subjected to during their preparation (e.g., shear stress, warm/cold ischemia, and nutrient depletion). Strikingly, after 96 h of incubation, slices cultured at 80% O_2_ accumulated even more DNA fragmentation over time, whereas DNA fragmentation in slices incubated at 20% O_2_ dropped to initial levels. The observed increase in DNA fragmentation at 80% O_2_ could be attributed to an increased production of reactive oxygen species (ROS), which can cause cell death if ROS production exceeds the capacity of anti-oxidant systems^28^. However, a downside of TUNEL stainings is that no inferences can be made regarding the molecular mechanism of cell death as both apoptotic and necrotic cells demonstrate DNA fragmentation. Furthermore, because the lungs contain over 40 different cell types, it was not possible to exactly determine what cell type survived or died^29^.

To determine whether caspase-dependent apoptosis was triggered in slices, we examined accumulation of its functional gene product cl-CASP3. Interestingly, cl-CASP3 gradually became more abundant in slices incubated at 20% O_2_, whereas at 80% O_2_ cl-CASP3 content remained unchanged over time. This finding indicates caspase-dependent apoptosis was responsible for cell death in lung slices cultured at 20% O_2_, although care should be taken not to overinterpret this finding because it does not mean other cell death mechanisms were not involved. Interestingly, recent studies have also implicated cl-CASP3 as an inducer of proliferation in neighboring cells, thereby triggering tissue regeneration^30^. Nevertheless, the lack of unchanged cl-CASP3 content in slices incubated at 80% O_2_ does suggest caspase-dependent apoptosis did not substantially contribute to cell death^31^. Instead, necrosis or caspase-independent apoptosis could have been considerably more prominent in slices cultured at 80% O_2_ because these processes are marked by a strong decline in ATP content, which we observed as well. The TUNEL staining supports this notion because DNA fragmentation, which results from apoptosis and necrosis, was more extensive in slices that were cultured at 80% O_2_^32^. Therefore, depending on the oxygen concentration, cell death was probably induced via different cell death mechanisms, which are neither isolated or mutually exclusive.

To estimate whether oxidative stress might have developed in slices, we assessed the transcription of NRF2-downstream anti-oxidant target genes (i.e., *Gclc*, *Gclm*, *Hmox1*, *Nqo1*, *Srxn1*, and *Txnrd1*). Typically, as a response to elevated ROS formation, NRF2 translocates to the nucleus, where it binds to anti-oxidant response elements, which are upstream of many anti-oxidant genes, including those tested in this study^21^. According to the results, mRNA expression of the tested NRF2-downstream target genes was more prominent in slices cultured at 80% O_2_, suggesting that the oxygen concentration affected the formation of ROS. Unfortunately, because the protein content in slices was too low, it was not possible to further investigate NRF2 accumulation in the nuclei of cells. However, an increased accumulation of NRF2 in the nucleus does not necessarily demonstrate the development of oxidative stress, which refers to an actual imbalance between the formation of oxidizing species and a cell its ability to detoxify the reactive intermediates. As such, our observations only demonstrated cells were strengthening their defenses to deal with potentially elevated ROS formation at higher O_2_ levels. Although extensive characterization of oxidative stress was beyond the scope of this study, future studies could consider the use of immunohistochemistry to detect nuclear accumulation of NRF2 and the measurement of oxidized and reduced glutathione to monitor oxidative stress^33^.

Furthermore, because necrosis can lead to acute inflammation, mRNA expression of pro-inflammatory cytokines was analyzed as well as the secretion of respective functional gene products. Over time, only *Il6* mRNA expression was elevated upon exposure to 80% O_2_. The discrepancy between *Il6* mRNA and IL-6 protein levels is likely caused by differences in their control mechanisms. IL-6 is stored in intracellular vesicles and can be rapidly secreted by cells, thus representing an acute response to injury^34^. Meanwhile, *Il6* mRNA expression in slices cultured at 80% O_2_ may have been induced via the nuclear factor κB (NF-κB) pathway due to persistent ROS formation, leading to differences between *Il6* mRNA and IL-6 protein levels. In addition, although variable over time, cytokine release was not dependent on the oxygen concentration. However, previously published results demonstrated a much stronger induction of *Il1b*, *Il6*, and *Tnfa* mRNA expression in slices incubated at 80% O_2_^3^. Importantly, in our previous study we used Accell siRNA delivery medium and in our current study we used PneumaCult-ALI medium, which contains hydrocortisone – a known immunosuppressive molecule. The presence of hydrocortisone (480 ng/mL) readily explains the observed suppression of acute inflammation^35^. It is certainly interesting to further characterize immunological properties of lung slices in terms of available immune cells and their phenotypes.

We subsequently investigated cell proliferation in slices by measuring mRNA expression of proliferation-related genes (i.e., *Mki67*, *Ccna2*, *Ccnb1*, *Ccnd1*, and *Ccne1*) as these genes are predominantly transcribed in proliferating cells^36,37^. Strikingly, mRNA expression of proliferation-related genes increased at 20% O_2_ and decreased at 80% O_2_. This highlights how the oxygen concentration not only affects cell death, anti-oxidant transcription, and acute inflammation but also cell proliferation. Perhaps, proliferation was impaired at 80% O_2_ due to AMPK-mediated activation of P53, which is known to induce cell-cycle arrest^38^. P53 can also be activated in cells upon exposure to severe stress and/or damage, such as DNA mutations, to prevent proliferation of abnormal cells^39^. Moreover, because expression of cyclins (i.e., *Ccna2*, *Ccnb1*, *Ccnd1*, and *Ccne1*) is strictly confined to specific cell-cycle transitions, our data clearly demonstrates cells in slices cultured at 20% O_2_ progressed through all cell cycles^39^. However, given that these findings are based on changes in mRNA expression, results should be treated with care as it is not possible to determine which and how many cells proliferated.

Finally, because differences in mRNA expression of proliferation-related genes were observed, we continued to check the localization of proliferating cells by staining for Ki67, which is a marker of cell proliferation^23^. After 48 and 96 h of incubation, slices incubated at 20 and 80% O_2_ displayed substantial differences in Ki67 staining intensity. More specifically, at 20% O_2_ Ki67 was abundant throughout the entire slices (i.e., airways and parenchyma), indicating extensive cell proliferation, whereas at 80% O_2_ Ki67 remained mostly confined to the airways. Furthermore, our Ki67 staining results are in line with mRNA expression data of proliferation-related genes as we observed substantial oxygen-dependent effects as well. Cell turnover in the lungs is normally very low, though injury can induce proliferation of progenitor cell populations (e.g., basal cells, airway secretory Club cells, and alveolar epithelial type 2 cells) to repopulate damaged areas^40,41^. As a result, these cell populations probably proliferated in slices. Nevertheless, further research is necessary to confirm this hypothesis. To that end, flow cytometry could be used to obtain both qualitative and quantitative insights into cell proliferation in slices.

## Conclusion

The effect of oxygen concentration (20 *vs*. 80% O_2_) on the extent of cell death, anti-oxidant transcription, acute inflammation, and cell proliferation in lung slices was investigated. The presented findings clearly illustrate lung slices cultured at 20% O_2_ displayed less cell death, anti-oxidant transcription, and acute inflammation, as well as more proliferation, demonstrating these slices were significantly more viable than slices incubated at 80% O_2_. We are confident that these findings expand our knowledge on lung slices and their optimal culturing conditions as well as their use as an alternative experimental model in drug research.

## Supplementary information

Supplementary information

## Data Availability

The datasets generated and analyzed are available from the corresponding author on request.
