# Supplementary material for: The effects of oxygen concentration on cell death, anti-oxidant transcription, acute inflammation, and cell proliferation in precision-cut lung slices
Source: Sci Rep. 2019 Nov 7;9:16239. doi: 10.1038/s41598-019-52813-2 (PMC6838147; doi:10.1038/s41598-019-52813-2)
Supplement: Supplementary file 1 — Supplementary information [file 41598_2019_52813_MOESM1_ESM.pdf]

The effects of oxygen concentration on cell death, anti-oxidant transcription, acute inflammation, and cell proliferation in precision-cut lung slices

Mitchel J.R. Ruigrok[1], Jasmine Tomar[1], Henderik W. Frijlink[1], Barbro N. Melgert[2,3], Wouter L.J. Hinrichs[1*], Peter Olinga[1].

[1]University of Groningen; Groningen Research Institute of Pharmacy; Department of Pharmaceutical Technology and Biopharmacy; Antonius Deusinglaan 1; 9713 AV Groningen; The Netherlands.

[2]University of Groningen; Groningen Research Institute of Pharmacy; Department of Pharmacokinetics, Toxicology, and Targeting; Antonius Deusinglaan 1; 9713 AV Groningen; The Netherlands.

[3]University of Groningen; Groningen Research Institute for Asthma and COPD; Hanzeplein 1; 9713 GZ Groningen, The Netherlands.

[*]Corresponding author (phone number: +31 (0)50 36 32398, e-mail address: w.l.j.hinrichs@rug.nl).

Supplementary information

Section 1 - Morphology scoring system

Introduction

Within the context of tissue damage, this protocol was used to assign morphological scores to hematoxylin and eosin (H&E) stained sections prepared from precision-cut lung slices. The airways and parenchyma were evaluated separately. Scores ranged from 0 (no tissue damage) to 4 (severe tissue damage) and, if applicable, were rounded to the nearest half (e.g., 2.5 or 3.5). Scoring was performed in a blinded manner by two independent observers, and Cohen's weighted κ was calculated to determine the inter-observer agreement ($\kappa_{airways}$ = 0.348 and $\kappa_{parenchyma}$ = 0.559). In addition, correlation analyses revealed a significant positive correlation between the two observers (fig. S1). The next two sub-sections will present scoring criteria as well as sample figures.

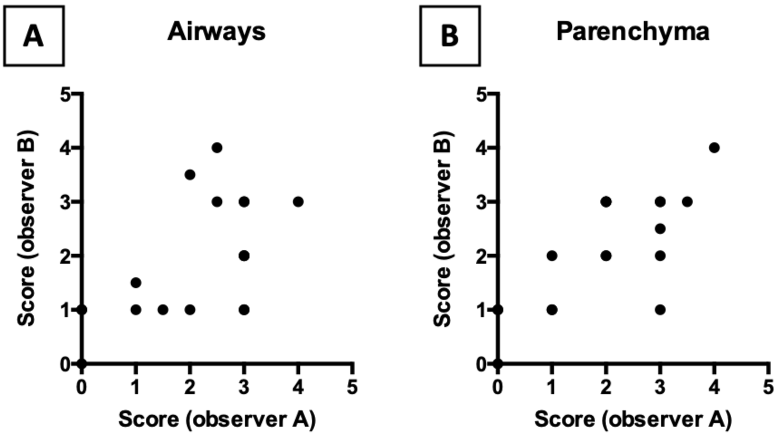

C

Section	Spearman r	*P* value
Airways	0,5169	0,0281
Parenchyma	0,7146	0,0009

Figure S1. Correlation analysis. Morphology scores for the airways (a) and parenchyma (b) were assigned in a blinded manner by two independent observers. Subsequent correlation analyses (c) revealed significant positive correlations between scores assigned by observer A and B.

Airways

Airway scoring criteria (table S1) is accompanied with sample figures (fig. S2).

Table S1. Airway scoring criteria.

Score	Damage	Criteria
0	None	The epithelium is clearly pseudostratified and healthy. Cilia can be observed as well.
1	Minimal	Epithelial cells start to flatten but are still connected to the basement membrane. Nuclei start to become pyknotic. Cilia may still be present at this stage.
2	Minor	Some epithelial cells start to lack nuclei. Cells are still connected to the basement membrane and cilia might still be observable.
3	Moderate	Aside from losing nuclei, epithelial cells start to detach from the basement membrane. Cilia are no longer present.
4	Severe	Nearly all epithelial cells have detached from the basement membrane. Cells that are still attached become strongly flattened to cover the remaining basement membrane.

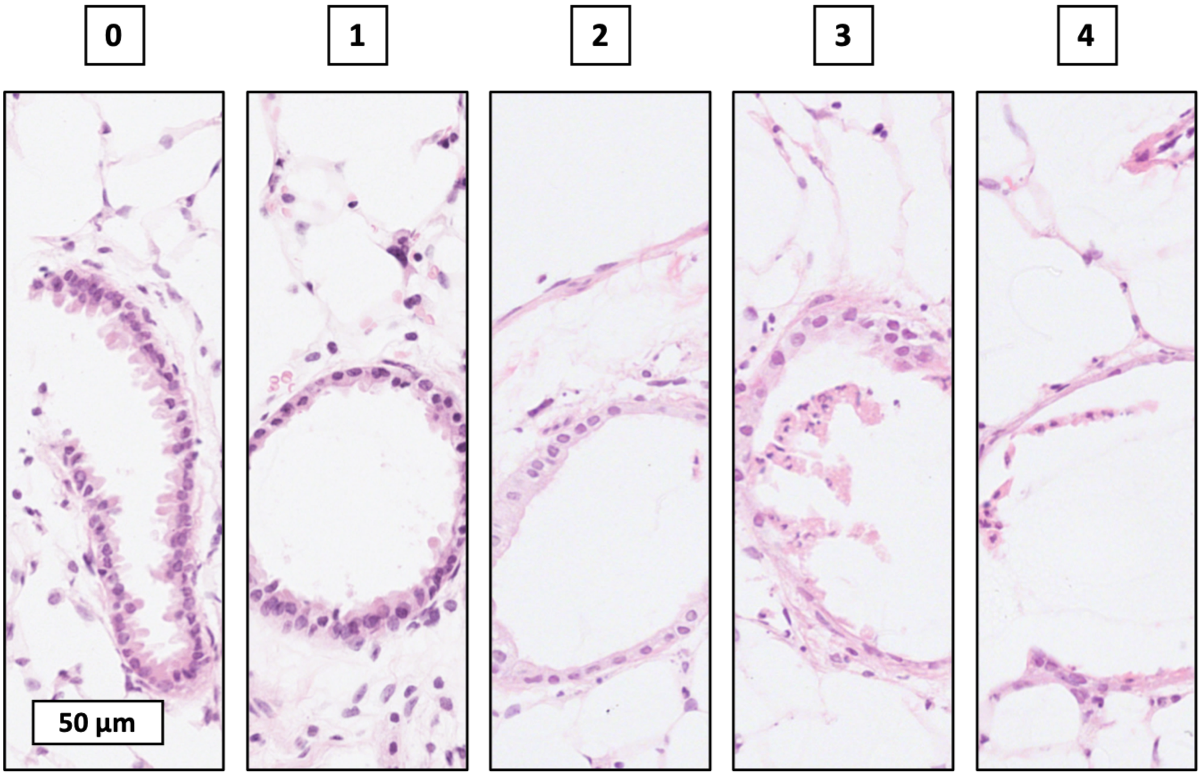

Figure S2. Airways. Morphology scores reflected no (0), minimal (1), minor (2), moderate (3), or severe (4) tissue damage in the airways.

Parenchyma

Parenchyma scoring criteria (table S2) is accompanied with sample figures (fig. S3).

Table S2. Parenchyma scoring criteria.

Score	Damage	Criteria
0	None	The tissue looks healthy and there are no signs of damage. Furthermore, erythrocytes may be observed.
1	Minimal	A small number of cells become pyknotic. These cells can be distinguished from normal nuclei as they are dark and small.
2	Minor	As apoptosis progresses, some apoptotic bodies can be observed. In addition, pyknosis is becoming more prevalent in the tissue.
3	Moderate	The tissue is now characterized by the clear presence of apoptotic bodies. Furthermore, pyknosis occurs throughout the entire tissue.
4	Severe	In this stage, a huge number of apoptotic bodies can be observed. The apoptotic bodies tend to cluster. Regions with no nuclei are clearly visible.

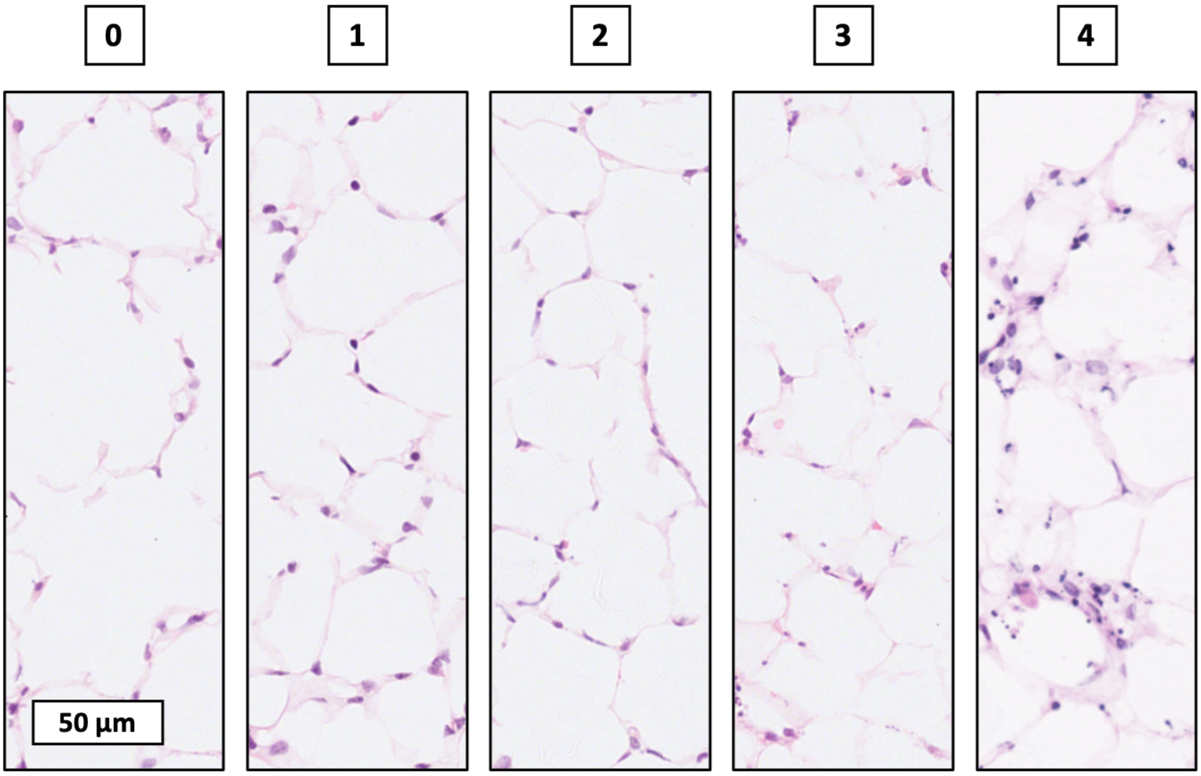

Figure S3. Parenchyma. Morphology scores reflected no (0), minimal (1), minor (2), moderate (3), or severe (4) tissue damage in the parenchyma.

Section 2 – Aperio Positive Pixel algorithm

The Aperio Positive Pixel algorithm (V9) is often applied to quantify a specific stain in scanned whole-slide images, based on certain color specifications. Default algorithm settings can be used to detect brown (DAB) and red (e.g., NovaRED) stainings against a blue (hematoxylin) stained background. In addition, the algorithm can be used to distinguish specific staining from non-specific staining when the staining intensity is calculated as the ratio of strong positive pixels *vs.* total pixels (fig. S4). This technical feature was very useful when analyzing Ki67-stained sections as airway epithelial cells displayed minor non-specific staining, whereas other cell types did not.

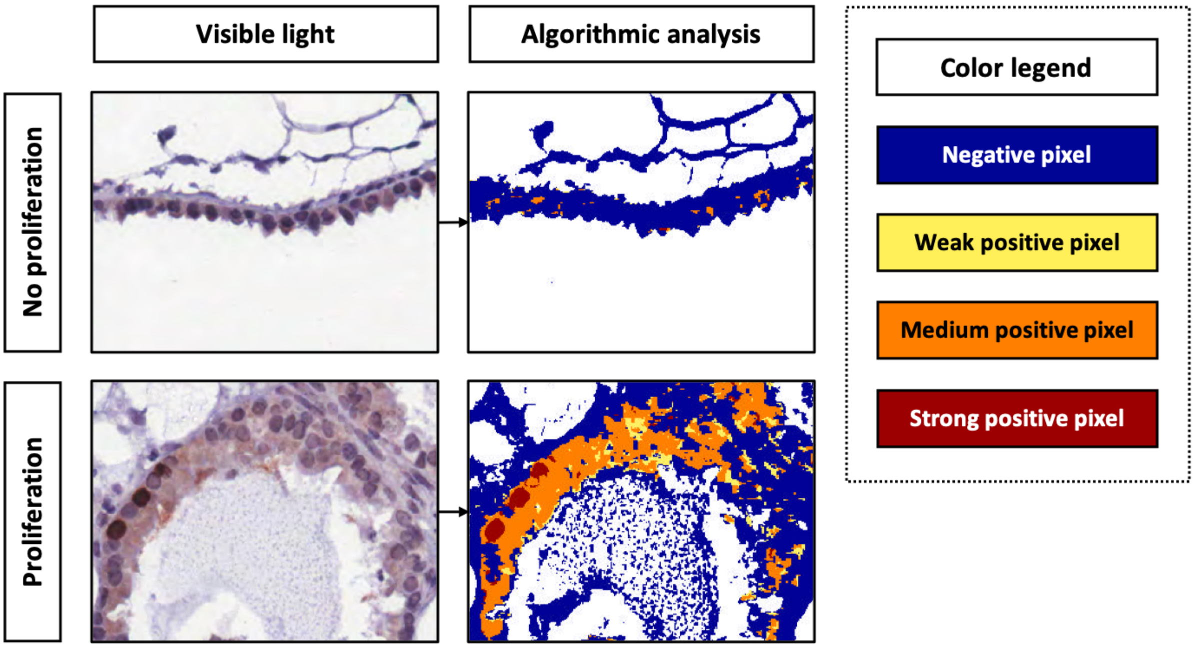

Figure S4. Aperio Positive Pixel algorithm. The default Aperio Positive Pixel algorithm (V9) can be applied to avoid detection of non-specific staining in the airways.
